# Supplementary figures and images for: SigRescueR: a pan-system framework for noise correction and mutational signature identification across sequencing platforms
Source: Brief Bioinform. 2026 Mar 6;27(2):bbag099. doi: 10.1093/bib/bbag099 (PMC12963972; doi:10.1093/bib/bbag099)

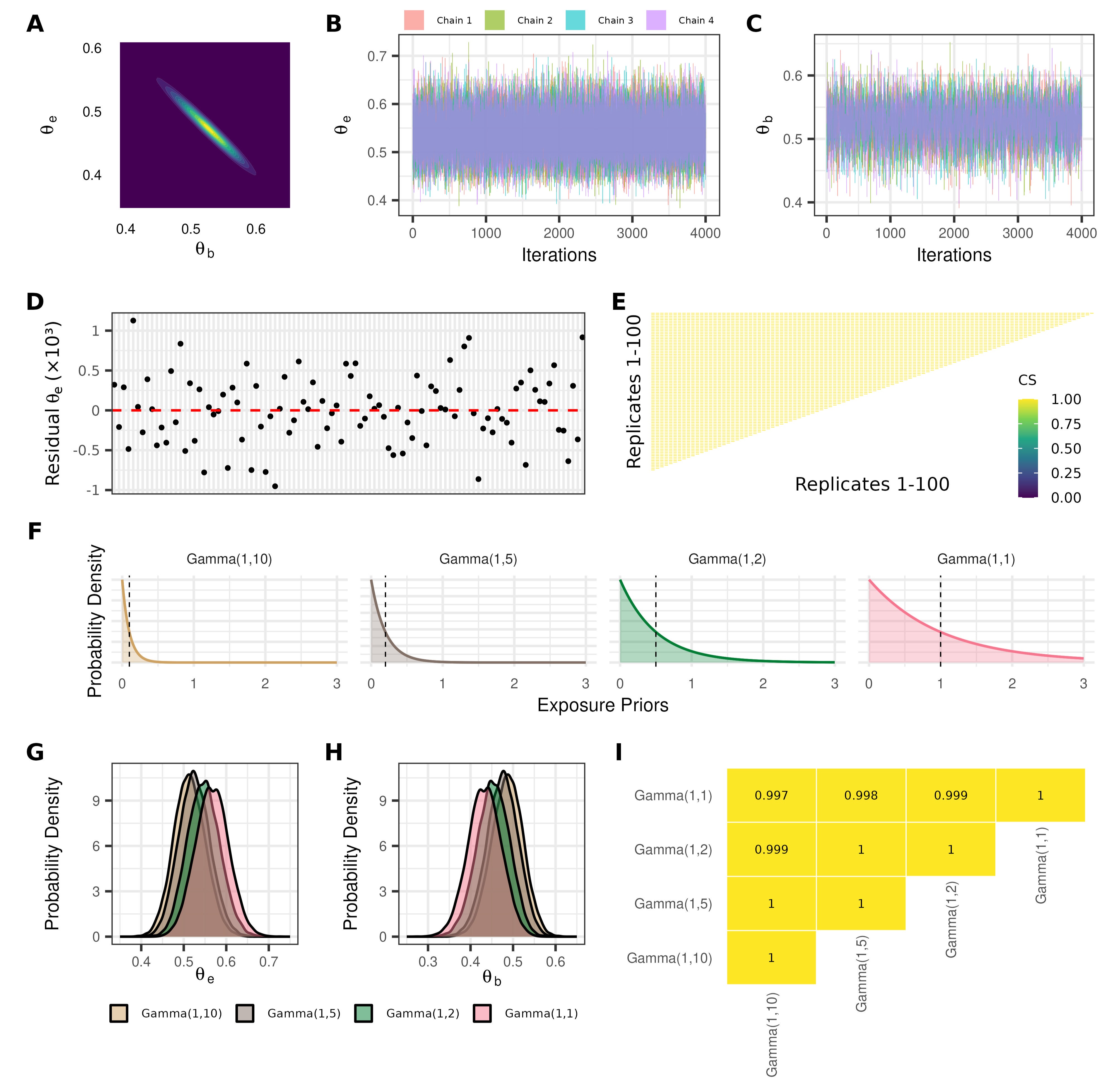

Supplement: bbag099_Supplemental_Files [file bbag099_supplemental_files.zip › suppfigure1_updated_bbag099.JPG]

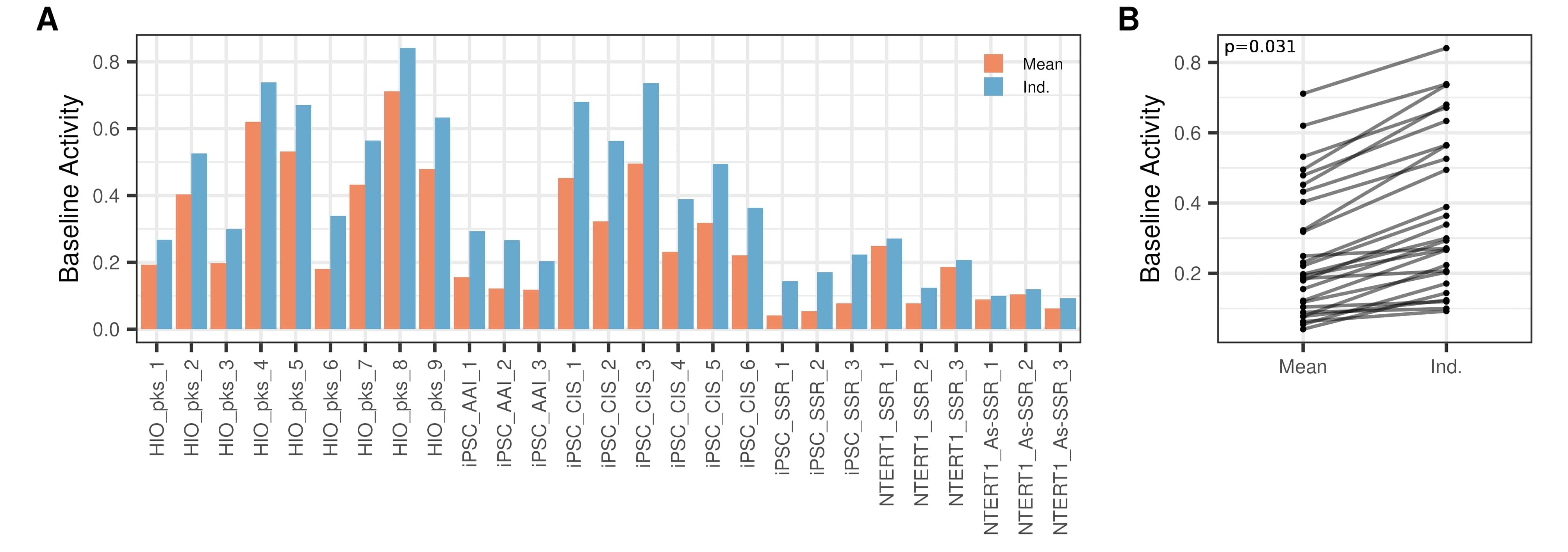

Supplement: bbag099_Supplemental_Files [file bbag099_supplemental_files.zip › suppfigure2_bbag099.JPG]

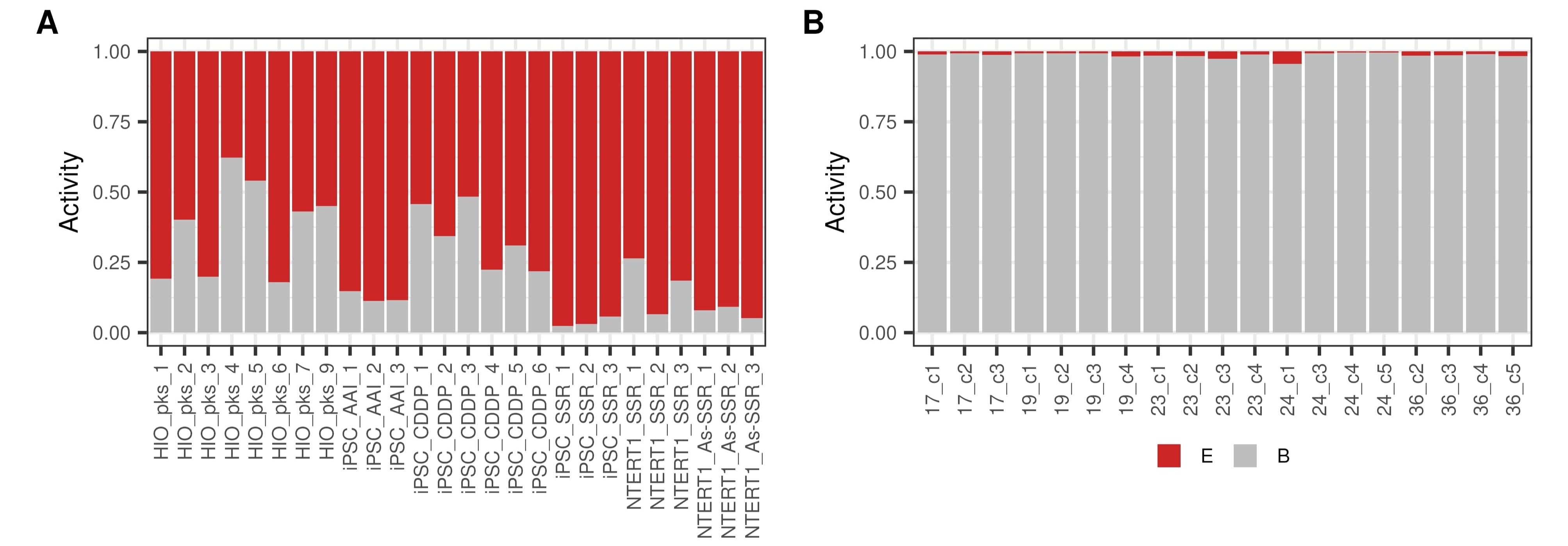

Supplement: bbag099_Supplemental_Files [file bbag099_supplemental_files.zip › suppfigure3_bbag099.JPG]

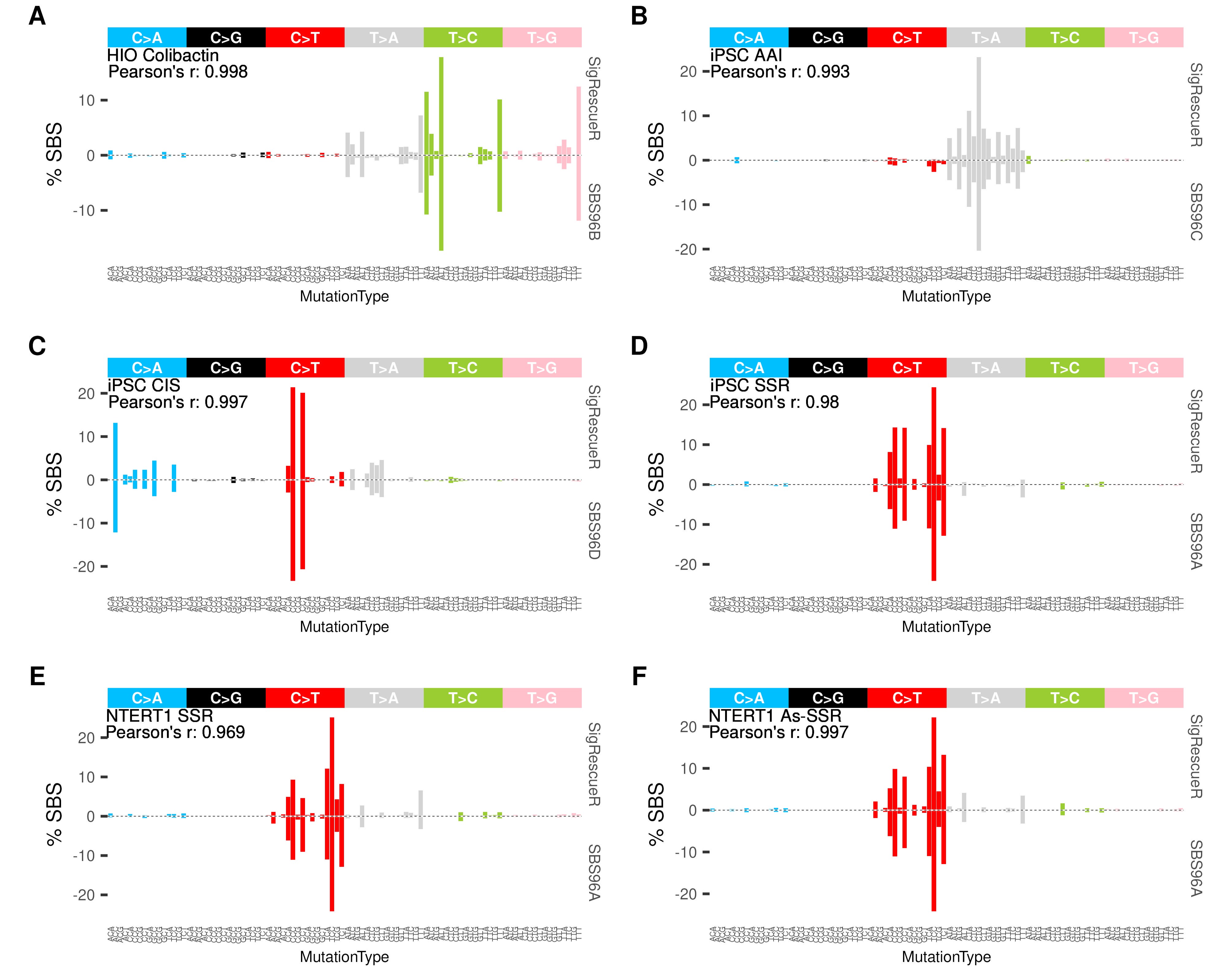

Supplement: bbag099_Supplemental_Files [file bbag099_supplemental_files.zip › suppfigure4_updated_bbag099.JPG]

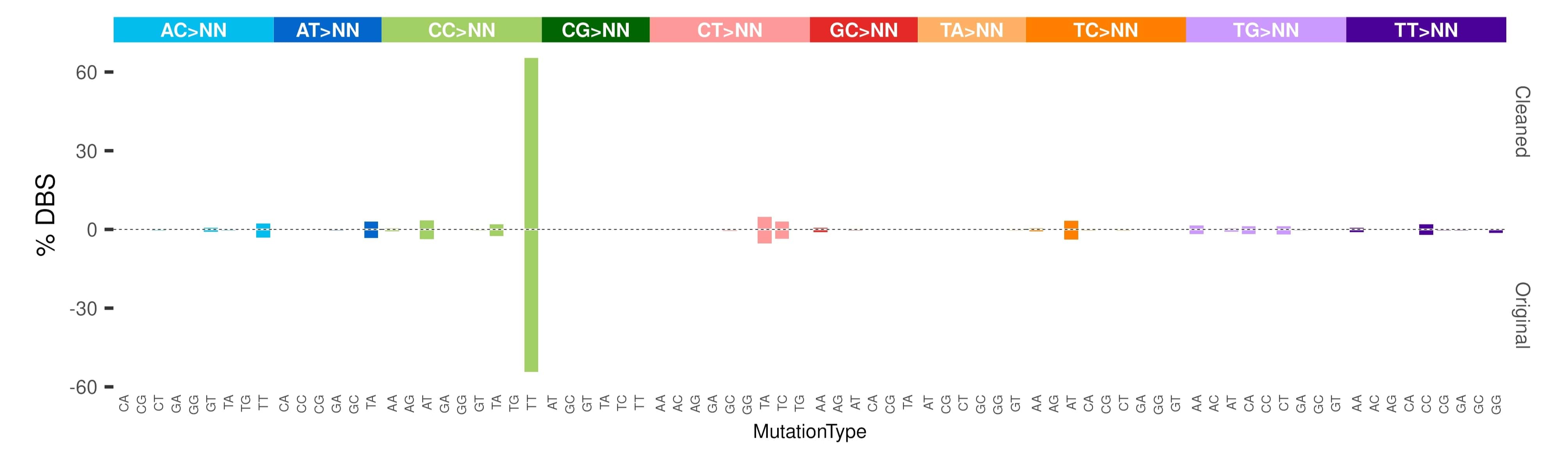

Supplement: bbag099_Supplemental_Files [file bbag099_supplemental_files.zip › suppfigure5_bbag099.JPG]

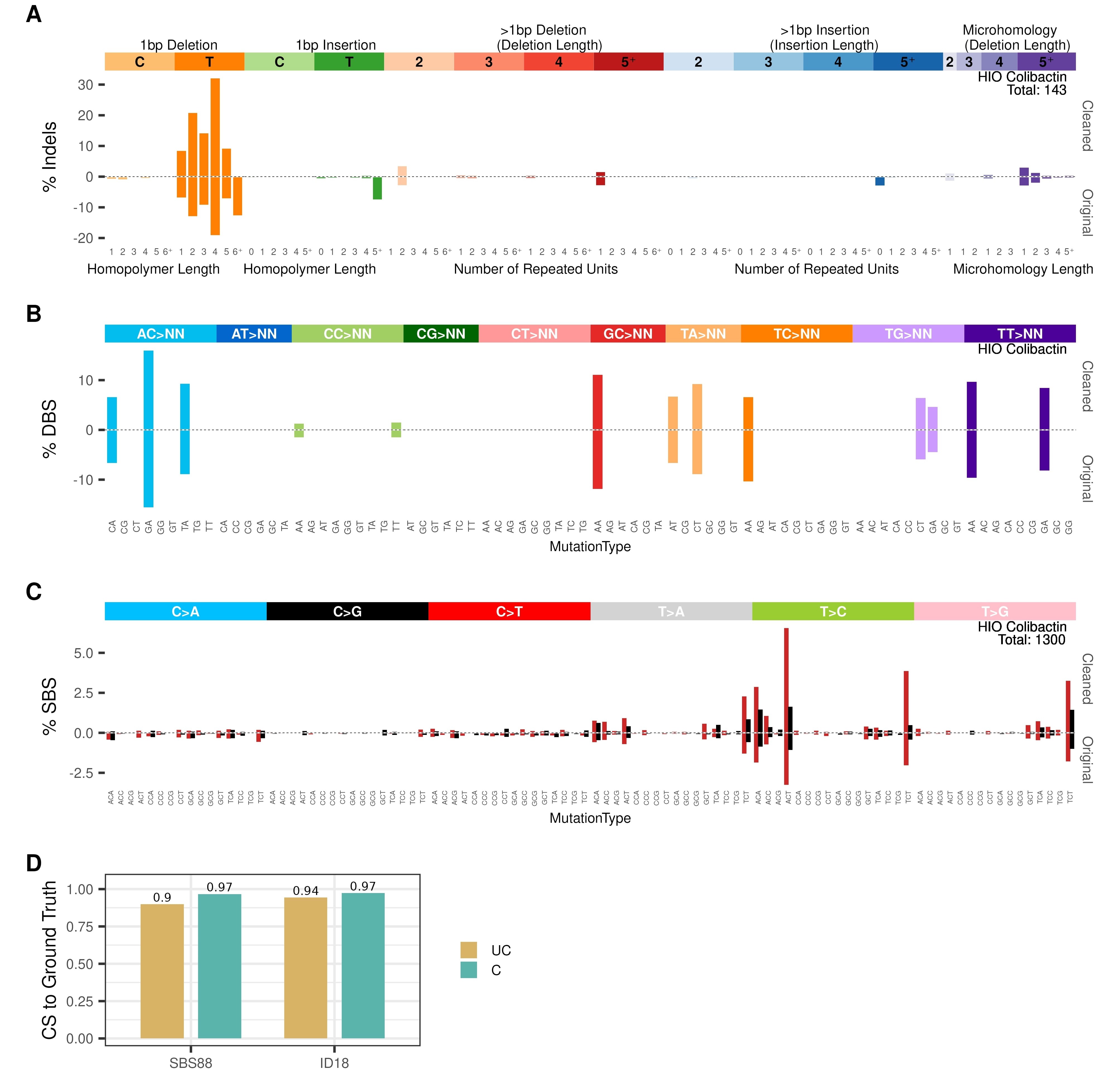

Supplement: bbag099_Supplemental_Files [file bbag099_supplemental_files.zip › suppfigure6_bbag099.JPG]

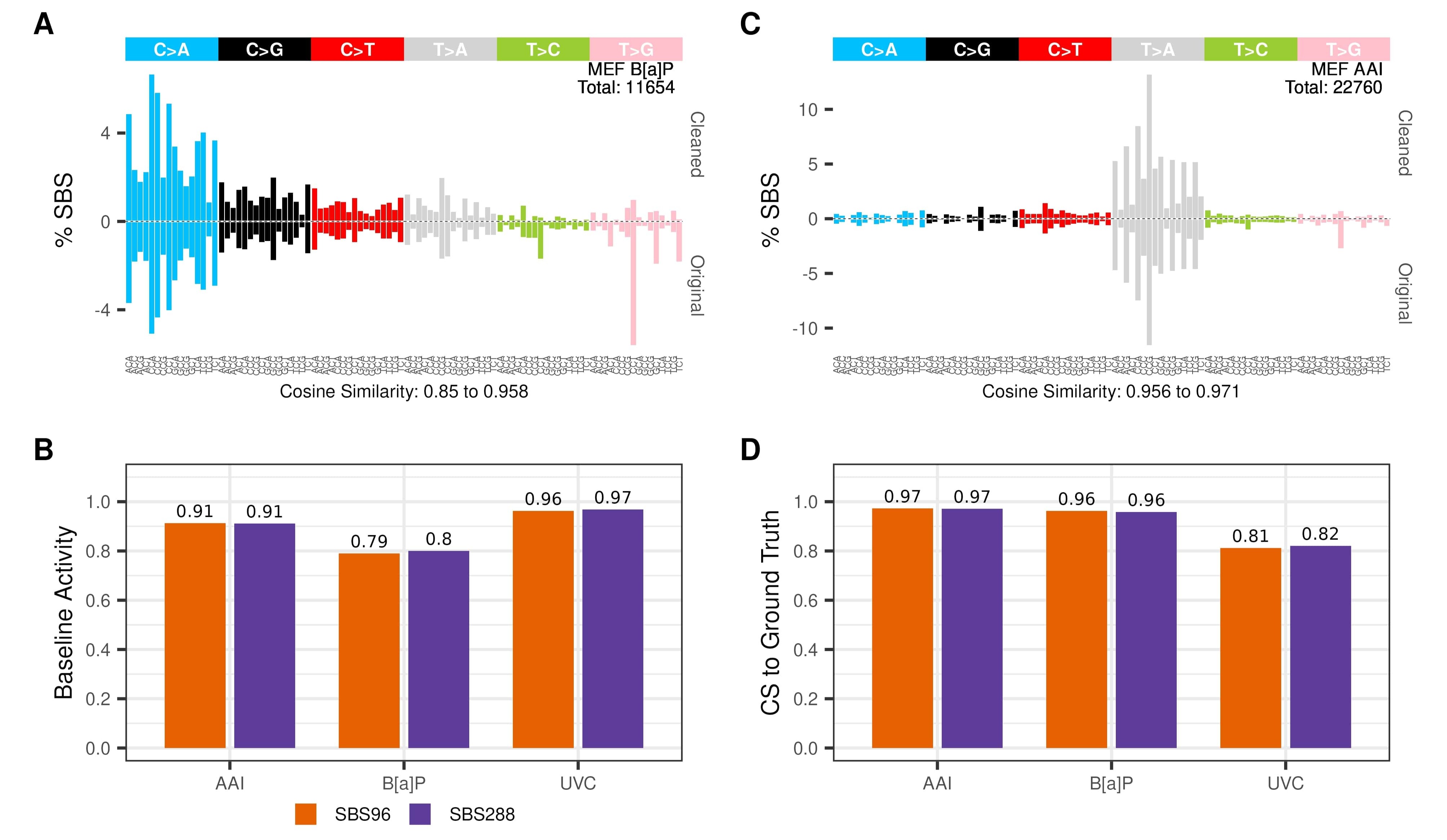

Supplement: bbag099_Supplemental_Files [file bbag099_supplemental_files.zip › suppfigure7_bbag099.JPG]

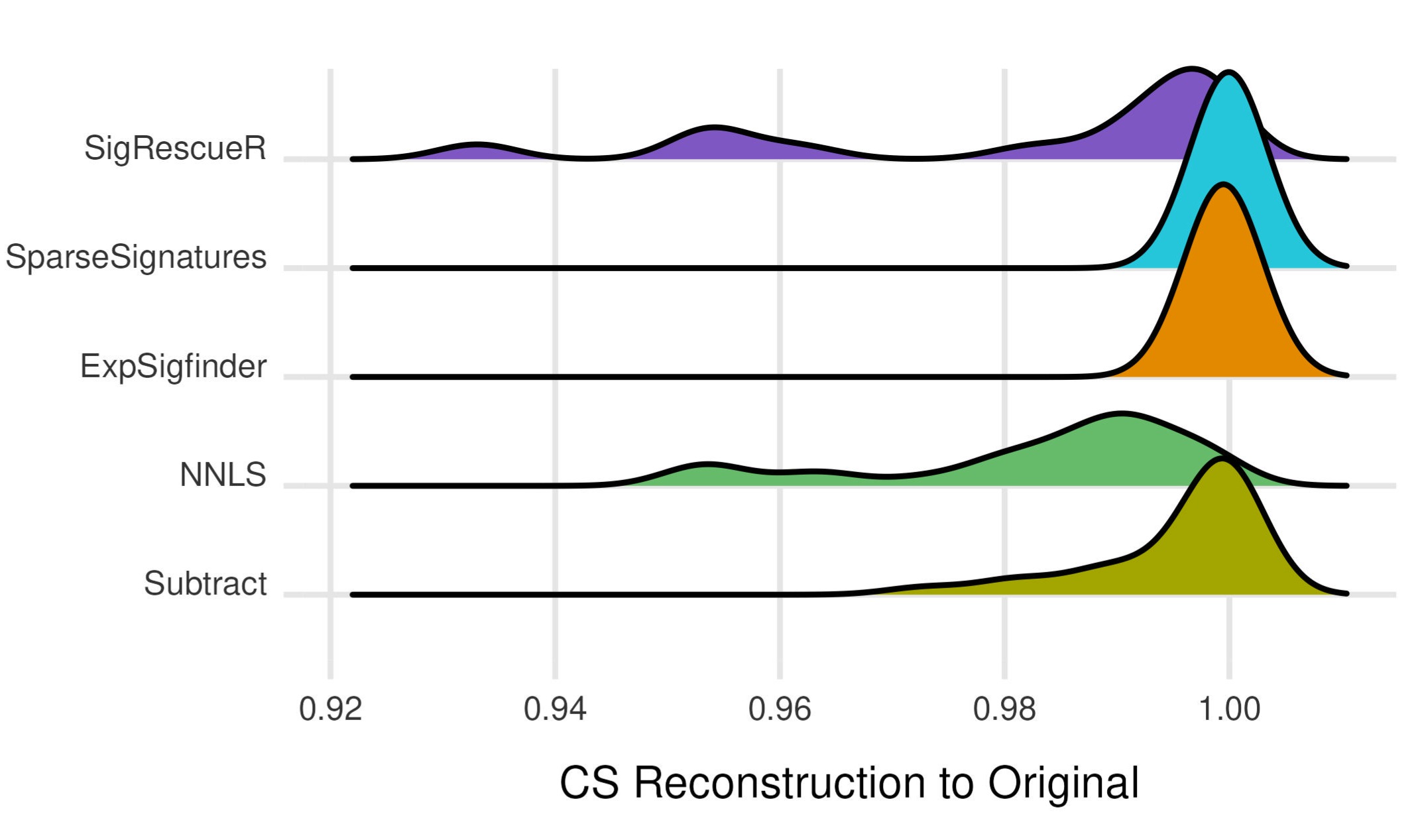

Supplement: bbag099_Supplemental_Files [file bbag099_supplemental_files.zip › suppfigure8_updated_bbag099.JPG]

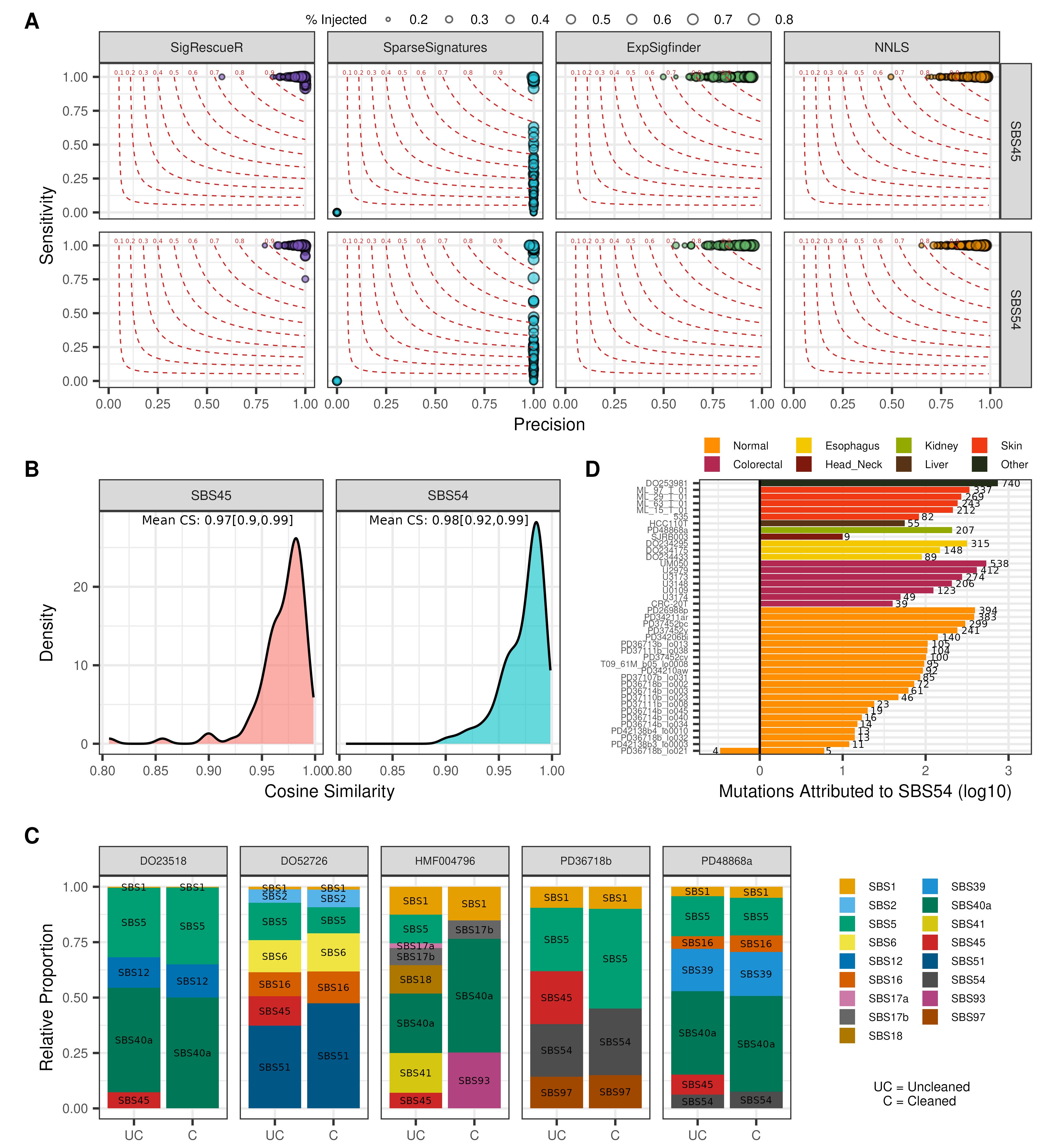

Supplement: bbag099_Supplemental_Files [file bbag099_supplemental_files.zip › suppfigure9_updated_bbag099.JPG]
